# Adaptation of the normative rating procedure for the International Affective Picture System to a remote format

**DOI:** 10.1186/s41155-024-00326-x

**Published:** 2024-09-27

**Authors:** Thayane C. Lemos, Laiz A. A. Silva, Sara D. J. Gaspar, Guilherme M. S. Coutinho, Jasmin B. Stariolo, Pedro G.M.R Oliveira, Lethicia S. Conceicao, Eliane Volchan, Isabel A. David

**Affiliations:** 1 Biomedical Institute, Universidade Federal Fluminense, Niterói, RJ Brazil; 2 Institute of Food and Nutrition, Universidade Federal do Rio de Janeiro, Macaé, RJ Brazil; 3 Instituto de Biofisica Carlos Chagas Filho, Universidade Federal Do Rio de Janeiro, Rio de Janeiro, Brazil

**Keywords:** Remote experiment, Online, Affective picture, IAPS, Emotion, Valence, Arousal, Self-Assessment Manikin

## Abstract

**Background:**

The Self-Assessment Manikin (SAM), a pictorial scale for the measurement of pleasure and arousal dimensions of emotions, is one of the most applied tools in the emotion research field.

**Objective:**

We present a detailed description of a remote method to collect affective ratings in response to pictures by using the SAM scale.

**Methods:**

To empirically validate our remote method, we conducted a study using a digitized version of the SAM scale and delivered online didactic instructions that followed the normative rating procedure for the International Affective Picture System (IAPS) to the participants. We presented 70 pictures from the IAPS and an additional set of 22 food pictures to the participants.

**Results:**

We found strong correlations between the ratings of IAPS pictures obtained in our sample and those reported by North American and Brazilian participants in previous in-person studies that applied the same pictures and methodology. We were also able to obtain an additional standardized set of food pictures.

**Conclusion:**

The protocols described here may be useful for researchers interested in collecting remotely valid and reliable affecting ratings.

**Supplementary Information:**

The online version contains supplementary material available at 10.1186/s41155-024-00326-x.

## Introduction

The development of replicable studies on emotional processing requires standardized visual stimuli. The identification of stimuli that differ quantitatively in their affective characteristics and can be easily manipulated by the experimenter is a priority goal in laboratory research on emotions (Quigley et al., 2014). The visualization of pictures with emotional content is one of the most commonly used procedures in emotion research because it meets some of these requirements (Cuthbert et al., 2000). The International Affective Picture System (IAPS) was developed at the Center for the Study of Emotions and Attention at the University of Florida (Lang et al., 2008). The goal of the IAPS is to offer calibrated emotional visual stimuli that can serve as standard stimuli, encouraging scientific replication and comparison in emotion research. Therefore, the IAPS catalog allows for a systematic and controlled study of emotions. pictures from the IAPS catalog are used in laboratories worldwide for the evocation of emotions (Branco et al., 2023), possibly replicating similar real-life emotions but in a controlled environment.

The construction of the IAPS was guided by Lang and Bradley’s theoretical model of the two dimensions of emotions: valence and arousal (Bradley et al., 2001). Motivational characteristics of behavior, such as approach to appetitive stimuli and avoidance of aversive stimuli, can be represented as quantifiable parameters of the pleasantness/unpleasantness (valence) and intensity of emotional activation (arousal), which define a hypothetical two-dimensional affective space (Bradley et al., 2001; Lang & Bradley, 2008). The process of validating the pictures from the IAPS involves applying a self-report method, the Self-Assessment Manikin (SAM) scale (Bradley & Lang 1994). The SAM uses nonverbal pictographic scales that are easy and quick to apply. The SAM provides information on two emotional dimensions: valence and arousal. The first dimension measures the degree of pleasantness of the picture, whereas the second dimension indicates the level of emotional alertness, activation, or arousal (Bradley & Lang, 1994). The participants rated each picture according to its emotional valence and arousal. There are nine levels for each dimension (valence and arousal) presented as a row of five manikins separated by blank spaces. The manikin expressions ranged from “smiling-happy” (score = 9) to “frowning-unhappy” (scoring = 1) for the valence dimension. The manikins’ emotions ranged from an “excited wide-eyed” figure (score = 9) to a “relaxed-sleepy” figure (scoring = 1) for the emotional arousal dimension (Bradley & Lang 1994). When the mean valence and arousal rating of each picture are plotted on a Cartesian plane, they are arranged in vectors pointing in two directions and represented by a “boomerang” shape, which configures the typical shape of the two-dimensional affective space (Bradley et al., 2001). The upper arm of the boomerang indicates appetitive motivation (“approach-like”), and the lower arm indicates aversive motivation (“avoidance-like”).

Hundreds of North American college students rated the pictures that comprised the IAPS on these two emotional dimensions by applying the SAM. The official normative ratings of the pictures from the IAPS were based on the reports of these North American participants as a function of the mean values of valence and arousal per picture (Lang et al., 2008). A key feature of the standardized affective pictures contained in the IAPS is the evocation of similar emotional responses across groups of individuals and cultures, allowing the comparison of findings from multiple laboratories worldwide. Studies conducted in different countries using SAM classification of IAPS pictures into valence and arousal resulted in similar ratings distribution of IAPS pictures in the affective space, with small variations (see Branco et al., 2023 for review). Additionally, by applying the same procedures used to generate the IAPS normative ratings, it is possible to create a new set of standardized pictures. For example, the IAPS methodology has been used to standardize catalogs of food pictures (David et al., 2018; Lemos et al., 2022; Miccoli et al., 2014) and pictures of social interaction (Silva et al., 2017) to be available for psychophysiological studies of emotion. The procedure consisted of presenting the “new” set of pictures, e.g., food or social interaction pictures, in addition to pictures from the IAPS, as background.

To ensure the reliability and validity of the affective ratings obtained with a new sample of participants and/or new pictures, experimenters must follow instructions from the normative rating procedure manual used in the original study with North American participants conducted by the research group at the University of Florida (Lang et al., 2008). In the original study, participants observed pictures that varied in valence and arousal and rated each of them using a pencil-and-paper version of the SAM. The experiments were conducted in a room containing a group of participants, and the pictures were projected onto a screen. The experimenters followed the experimental instructions and sessions in-person (Lang et al., 2008). Although this method may have proven extremely valid, in-person application of the protocol applied to the IAPS may have some limitations, for example, requiring participants to be physically present may discourage participation, as it adds financial and time costs related to traveling. Experimenters also have less flexibility in timing and space when the experiment occurs face to face. Consequently, experiments are often conducted in university classrooms, which limits sample diversity. Remote data collection can minimize these logistical problems, favoring the participation of more individuals, including those with mobility problems or those located in different regions of the country or even in different countries. Remote data collection became even more important in the context of the COVID-19 pandemic, where individuals were confined to their homes. Researchers in experimental psychology have developed creative solutions using remote data collection to conduct their experiments (Shin et al., 2021; Weydmann et al., 2022).

Here, we aimed to test the validity of a remote experiment conducted using the protocol applied for the IAPS. The reliability and validity of the affective ratings obtained with remote data collection in our study will be tested by attempting to replicate the findings from the normative ratings obtained in the original study with North American participants (Lang et al., 2008). We expect to replicate the typical boomerang-shaped distribution of picture ratings within the bidimensional (valence-arousal) affective space. We also hypothesized that the remotely collected valence and arousal ratings in our sample correlate strongly with the valence and arousal ratings from the original North American study with data collected in-person by the experimenters. Furthermore, we also aimed to examine the correlation between the valence and arousal ratings for the IAPS pictures acquired from our sample and those obtained from another study that collected data from a sample of Brazilian university students in-person (Lemos et al., 2022).

Finally, we also evaluated whether remote data collection enabled obtaining an additional standardized set of pictures for use in psychophysiological studies. Accordingly, we used a new set of food pictures that are not part of the IAPS catalog and that were used in a previous study where the valence and arousal ratings were collected in-person by the experimenters using the same methodology applied for the IAPS (Lemos et al., 2022). We expect the valence and arousal ratings obtained for this additional set of food pictures to be associated with the ratings previously obtained for these same pictures in the study by Lemos et al. (2022).

## Materials and methods

### Participants

Three-hundred and sixty-four university students from the Federal University of Rio de Janeiro (Brazil) volunteered to participate in the study. They were recruited via email, social media, or classroom visits. Individuals who were aged between 18 and 30 were native Portuguese speakers, had access to a computer or notebook with Internet connectivity, were omnivores, and reported normal or corrected visual acuity were added to the experimental sample. The exclusion criteria included having a diagnosed eating disorder, being pregnant, and evaluating less than 50% of the pictures presented. The final sample consisted of 247 university students enrolled in various fields of study (biological/health sciences, natural sciences, mathematics, engineering, social sciences, linguistics, and humanities) who were naive to the study’s goal and had a median age of 22 years (interquartile range (IQR) = 4.00).

Sixty-seven percent of the participants were women (*n* = 167), 29.1% were men (*n* = 72) and 3.2% were nonbinary (*n* = 8). This gender profile matched that from Lemos et al. (2022), in which most of the sample was composed of women. Approximately half of the participants declared that they were white (54.7%, *n* = 135). Black/brown individuals represented 43.3% of the sample (*n* = 107), whereas the remainder (2%) reported being Asian/yellow or chose not to report their race. The mean body mass index (BMI) of the participants was 23.7 kg/m^2^ (standard deviation (SD) = 4.6). Most of the individuals were eutrophic (59.9%, *n* = 148), according to the World Health Organization cutoff (WHO 2000). The local ethics committee approved the experiment (CAAE: 29,357,820.1.0000.5699), and all participants provided their consent before any experimental procedures were performed.

### Pictures

#### International Affective Picture System

In accordance with Lang et al. (2008), we chose 70 IAPS pictures with various affective contents to encompass representations of positive, negative, and neutral valences. In addition, each valence category had pictures with different levels of arousal. Thus, the pictures were chosen to prompt positive or negative emotional experiences varying in arousal intensity interspersed with pictures that were relatively neutral. The positive pictures included 10 pictures depicting nature, family, dogs, sports, adventure, and eroticism; the neutral pictures included 30 pictures depicting objects, people, and landscapes; and the negative pictures included 30 pictures depicting pollution, disgusting objects, illness, loss, accidents, contamination, animal attacks, human attacks, and mutilated bodies. The codes for all the IAPS pictures used can be found in the supplementary material 1. Five IAPS pictures in Lemos et al. (2022) were replaced in the present study. The following IAPS pictures were included in this study but not in the Lemos et al. (2022) study: 1710, 2070, 2165, 3130, and 4660 (Lang et al., 2008).

#### Additional set of food pictures

We were also interested in obtaining an additional standardized set of food pictures by using the remote version of the normative rating procedure for the IAPS. To this end, we included 22 food pictures (for a total of 32 positive pictures) along with the IAPS pictures. A previous in-person study that used the IAPS’s normative rating procedure demonstrated the validity and reliability of the affective ratings of this new set of food pictures (Lemos et al., 2022). The food pictures were the same as those in Lemos et al. (2022). The dataset was categorized into ultra-processed and unprocessed/minimally processed food, according to the NOVA classification system, which divides food into categories based on the type, degree, and goal of industrial processing (Monteiro et al., 2016). Half of the food pictures (*n* = 11) were from ultra-processed food, while the other half (*n* = 11) were from unprocessed/minimally processed food. The unprocessed/minimally processed food pictures included watermelon, apple, mandarin juice, salad, corn, egg, lettuce, pear, mango, banana, and bean. The ultra-processed food pictures included gum, potato chips, chocolate bar, ready-to-eat lasagna, ice cream, margarine, cookie, soft drinks, sausage, cookies stuffed with vanilla, and Brazilian cheese bread (Lemos et al., 2022).

For 164 of the participants, the pictures of the ultra-processed foods included a warning label as part of the goal of a different study that is not relevant to this one. As a result, the valence and arousal mean ratings computed for the pictures of the ultra-processed foods are based on the 83 participants (out of 247 total participants) who viewed these pictures without any warning labels.

### Experimental procedure

#### Tools for remote data collection

We utilized the software PsychoPy 3.0® version 2021.2.0 (Peirce et al., 2019) to adapt the experiment to a remote format while maintaining the control parameters required for the normative rating methodology applied for the IAPS (Lang et al., 2008). PsychoPy 3.0v® is a free software tool that allows a wide variety of behavioral science studies to be performed. With this software, behavioral studies can be carried out remotely without sacrificing quality. A script was created with HTML and JavaScript to display the visual stimuli in a browser by exporting the programmed experiment to a webpage (e.g., https://pavlovia.org/), which enabled the users to access the experiment with high temporal precision from any location. It enables participants to be managed online, for existing experiments to be cloned and altered, and experimental results to be managed using the pavlovia.org website (Peirce et al., 2019). We used Google Forms to collect information about sociodemographic characteristics. Using WhatsApp, the experimenters supervised the participants during the experimental session.

#### Sequence of events

**Fig. 1:**
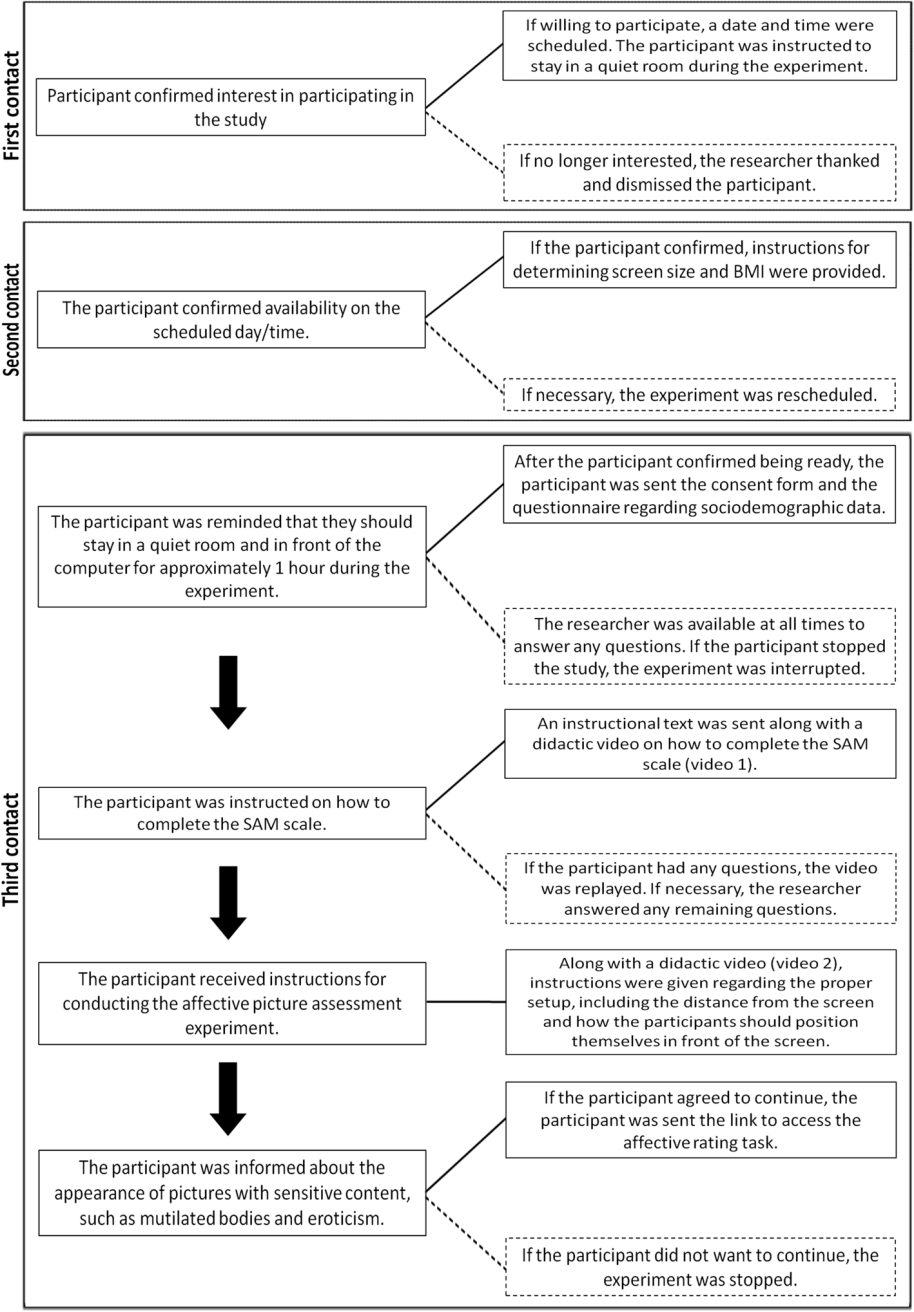
Flow chart showing the sequence of events and key points of decision-making during the study. Solid lines represent the typical sequence of step completion in the study. Dashed lines indicate the stop or restart of a step. Links for the didactic videos is as follows: http://labnec.sites.uff.br/2023/03/02/experimental_tools/. BMI, body mass index

Figure 1 displays a flow chart of the order of events. In the first phase of recruitment, the participants gave the experimenter their WhatsApp number, which served as a means of communication. An experimenter from our team addressed each participant and was in charge of conducting the experiment with them one-on-one. The first contact was followed by a standardized script prepared by our research team that consisted of three steps.

1. First, contact was made to ascertain interest in the study and to confirm access to a computer or notebook and the Internet. The participants were also instructed to stay in a silent room during the experiment and were asked to confirm their availability for the duration of the experiment.
2. Second, the participants were contacted the day before the study to confirm the scheduled date and to send instructions about the experiment (e.g., how to measure the computer screen’s size, and not to eat, talk, or engage in other activities during the experiment).
3. The research date was scheduled based on the participant’s availability to stay in an adequate environment during the experiment (e.g., being alone in a silent room with access to a computer and Internet). Once the participant was ready, the researcher emailed links to a sociodemographic information survey (using Google Forms) and the behavioral task (https://pavlovia.org), which involved the affective rating of the pictures using the SAM scale. The participants used a web browser to access the experimental task. This method eliminated the requirement for users to download customized software or apps, which may be perceived as more time-consuming and risky for security than using a web browser (Rodd, 2024). Before the behavioral task, instructional videos were also provided, outlining the procedures for classifying the pictures using the SAM scale.

#### Instructions

On the date prior to the scheduled date for the experiment, the experimenter sent a message via WhatsApp to instruct the participant to measure the screen size of the computer or notebook that would be used to perform the experiment and to provide information about weight and height (supplementary material 2). Body mass index (BMI) was calculated using the height and weight data, and it was used in a different study that will not be discussed here. Then, on the scheduled date, the experimenter contacted the participant again via WhatsApp to perform the experiment. The participants performed the entire procedure remotely on a personal computer or notebook. First, the participants received a link to access Google Forms, which started with the ethics consent form. After signing the ethics consent form electronically, sociodemographic data was collected. Then, the participants received a text with general instructions on how to fill out the SAM scale based on the “instructions for adults participants” provided in the IAPS instruction manual (Lang et al., 2008). The participants were shown a didactic video prepared by our research team that outlined the steps for completing the SAM scale after viewing each picture. The video (video 1) can be accessed at labnec.sites.uff.br/2023/03/02/experimental_tools/. Following completion of this step, the participant received instructional pictures (supplementary material 3) and another didactic video (video 2, labnec.sites.uff.br/2023/03/02/experimental_tools/) from the experimenter, reiterating the importance of using a computer or notebook for the experiment and positioning it in a quiet room to prevent distractions. They were also reminded to use eyeglasses or contact lenses to adjust their vision if needed and received instructions explaining that they needed to maintain a distance of approximately 47 cm from the computer screen (accompanied by pictorial examples, supplementary material 2) and to report the screen’s size [the mean reported computer screen size was 15.9″ (*SD* = 3.43)]. This allowed the experimenters to obtain information about the stimuli’s size in terms of visual angle. The pictures occupied approximately 47% of the screen’s area. The visual angle of the pictures was approximately 18.1° high and 26.3° wide, which was large enough to evoke emotional responses (De Cesarei & Codispoti, 2006).

Finally, once any participant doubts were addressed, the experimenter supplied a link to the pavlovia.org page containing the behavioral (affective rating) experiment.

### Experimental design

#### The Self-Assessment Manikin (SAM) scale

The SAM scale (Bradley & Lang, 1994), which was initially created in the paper–pencil version, was modified for the remote version. On the participant’s computer screen, the digitized SAM scale (size [height, width] ≅ 13.9°, 35.4°) was placed in the center of the screen. The method of rating involved clicking on the choice in each dimension (valence and arousal) that most accurately represented the participants’ emotional perceptions of each picture using a mouse or touch pad. To maintain the reliability of the emotional aspects assessed, unlike for sliders, the initial position of the cursor was arbitrary, and the cursor could be placed anywhere on the screen. The participants were limited to selecting a single response option for each dimension of the SAM scale. The participants were also able to not fill the SAM scale (skip responses), which could also occur, although not desirably, in the in-person experiments.

#### Trial structure

**Fig. 2:**
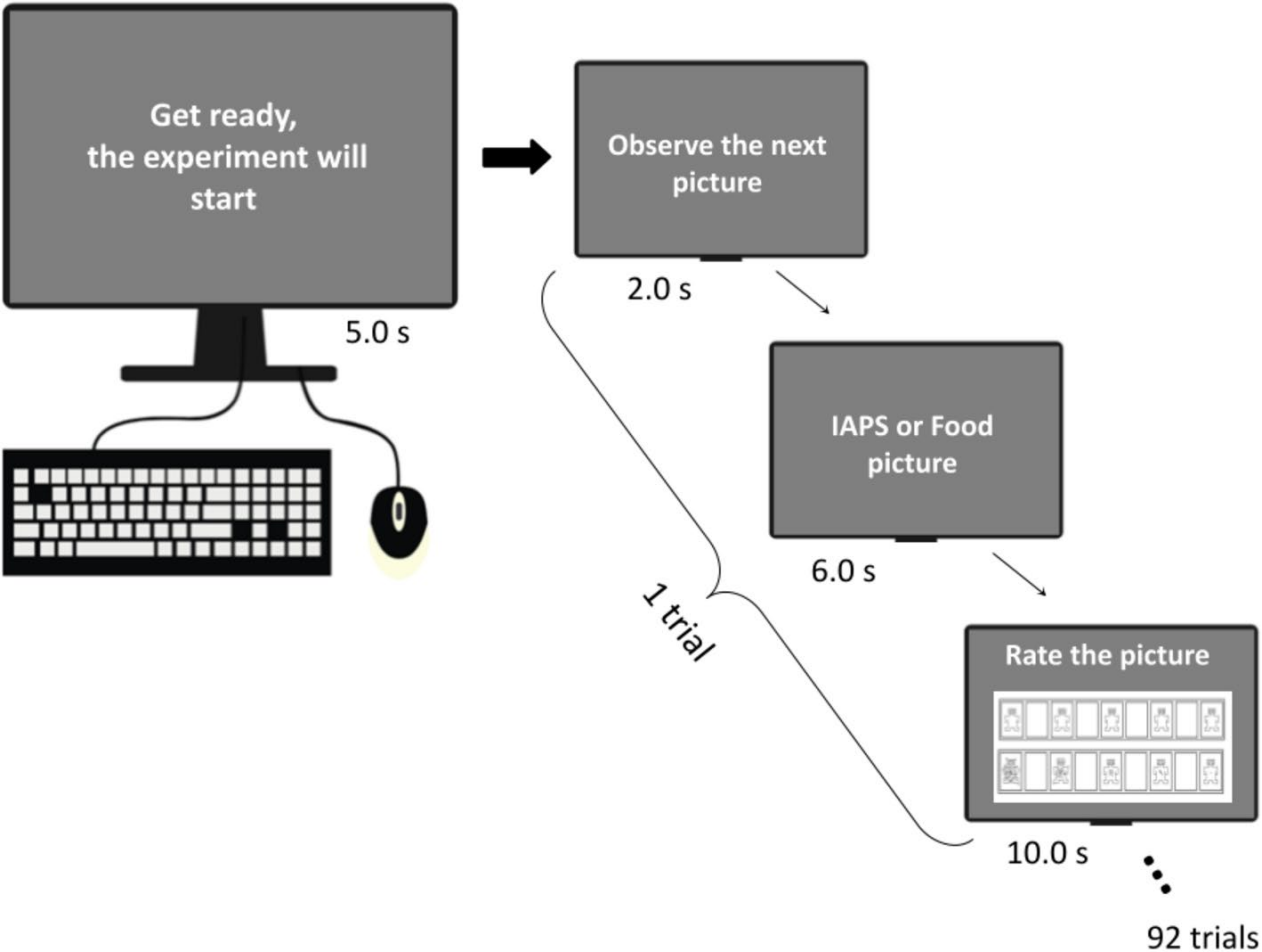
Schematic representation of the sequence of events in a trial. A total of 92 trials were presented, with each of the 22 food pictures and 70 IAPS pictures appearing randomly. Once the picture preview was finished, participants rated how they felt by clicking on the digital Self-Assessment Manikin scale

Before the experimental trial began, nine more IAPS pictures were utilized for training (three positive, three negative, and three neutral). This process provided the participant with an overview of the emotional content of the pictures that would be given during the experiment and served as an anchor for the affective ratings. Following the training, the participants were given the following instructions: “Click the mouse or touchpad to begin the experiment when you're ready.” After the mouse was clicked, the phrase “Get ready, the experiment is about to begin” was displayed on the screen for five seconds before the experiment started. This was the only message that appeared at the start of the experiment, and it was meant to get the participant ready. The sequence of events for each trial is described in Fig. 2. “Observe the next picture” was displayed on the screen at the start of each trial, lasting for 2 s. Afterward, the participant was shown a picture and had 6 s to focus solely on it. The picture was a food picture or a picture from another emotional category of the IAPS. After the picture, the “Rate the picture” screen appeared. The participants used the SAM scale (Bradley & Lang, 1994) to rate the presented picture in the valence and arousal dimension of emotion during the 10-s presentation of the final screen. The SAM scale screen vanished after 10 s, and a new trial started. These trial events were repeated until all 70 IAPS pictures and 22 food pictures were rated (Fig. 2).

### Data analysis

#### Distribution of the affective ratings in the bidimensional affective space of valence and arousal

The first step was to check whether the distribution of ratings in the two-dimensional affective space (valence-emotional arousal) fit the typical boomerang shape reported by Lang and colleagues in successive North American versions of the IAPS (Bradley & Lang, 1994; Lang et al., 2008). To this end, the values of valence (pleasantness) and emotional arousal for each picture were obtained by calculating the average of the valence and arousal values reported by the participants for each picture, and the averages were plotted on a Cartesian plane, with the “y”-axis representing valence and the “x”-axis representing arousal.

#### Correlations between the current study’s affective ratings and those from previous in-person studies

Spearman’s correlation was conducted in two steps: first, the association between the valence ratings from the IAPS pictures of the present study and those originally obtained in-person from North American university students (Lang et al., 2008) and from Brazilian university students (Lemos et al., 2022) was examined; second, the correlations of the emotional arousal data for these same samples were evaluated. These analyses did not include pictures 1710, 2070, 2165, 3130, or 4660 because they were not presented in the Lemos et al. (2022) study. The Bonferroni-adjusted *p*-value considered to indicate statistical significance was *p* < 0.025.

#### The additional set of food pictures

Spearman’s correlation test was applied to confirm the association between the ratings of the food pictures obtained in this sample and those reported by Lemos et al. (2022). Two analyses were performed separately: one for the valence ratings and the other for the arousal ratings. Additionally, we obtained the participants’ hunger ratings (Grand 1968) and converted them into a score (see Tapper & Turner 2018). We performed Spearman’s correlation between the food picture arousal and valence ratings obtained from the current remote study for each emotion dimension and the hunger scores. The Bonferroni-adjusted *p*-value considered to indicate statistical significance was *p* < 0.025.

## Results

### The boomerang-shaped distribution of the pictures in the bidimensional affective space

**Fig. 3:**
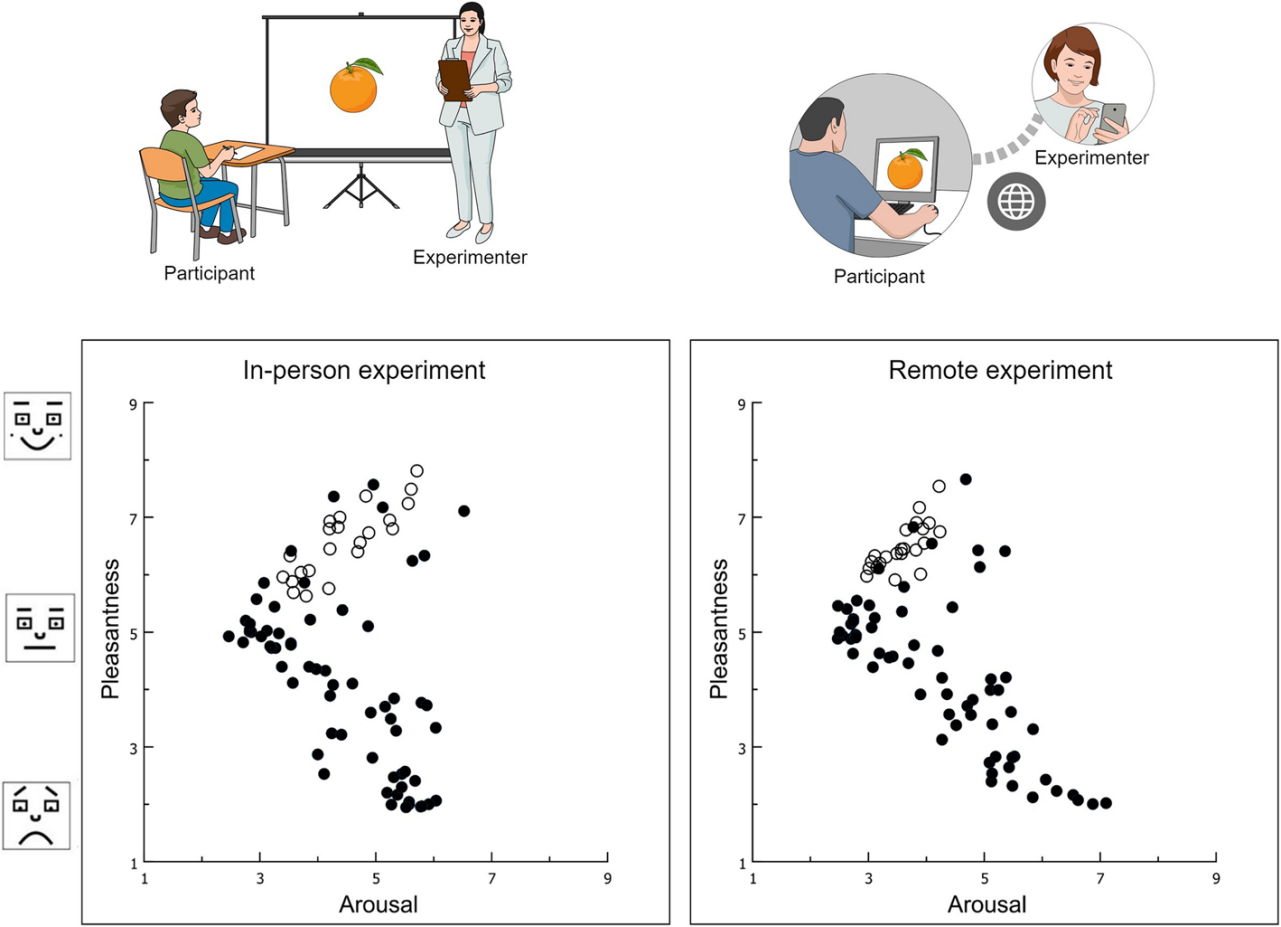
Plot in the two-dimensional affective space of each IAPS [black dots (●)] or food picture [white dots (○)] as a function of its mean valence (*y*-axis) and arousal ratings (*x*-axis) obtained during the Lemos et al. (2022) in-person study (left panel) and during the current remote study (right panel). A sample of Brazilian university students was used in both studies. Some pictures utilized in the current remote study were not plotted on the graph because they were different from those used in the Lemos et al. (2022) study. IAPS, International Affective Picture System

Figure 3 illustrates the distribution of the rating of the pictures in the two-dimensional affective space defined by the dimensions of valence (*y*-axis) and arousal (*x*-axis) for the current remote study (right panel) and for the study of Lemos et al. (2022) (left panel). The boomerang-shaped distribution of the pictures in the bidimensional affective space typically found in this type of study (Barke et al., 2012; Bradley et al., 2001; David et al., 2018; Miccoli et al., 2016; Silva, 2011; Soares et al., 2015) was reproduced (Fig. 3 right panel), indicating that the IAPS pictures were properly distributed in the affective space in our study. The pleasant pictures from the IAPS (e.g., erotic pictures, sports, families, nature, and puppies) were located in the upper half of the chart, while the unpleasant pictures from the IAPS (e.g., mutilation, illness, pollution, accidents, and disgusting pictures) were located in the lower half of the chart. The neutral pictures from the IAPS (objects) were located in the center of the graph.

### Pictures from IAPS: correlations between the current study’s affective ratings and those from previous in-person studies

Spearman’s correlation test was applied to confirm the association between the ratings of the IAPS pictures obtained in this sample (in which the data were collected remotely) and those reported by studies that carried out the experiment in-person (Lang et al., 2008; Lemos et al., 2022). These correlations allowed us to assess the validity of the methodology, determining whether it was applied correctly. There was a strong correlation between the data from the original in-person IAPS study with North American university students (Lang et al., 2008) and the data obtained from our sample collected remotely, both for valence (rho = 0.96, *p* < 0.001, Table 1) and arousal (rho = 0.85, *p* < 0.001, Table 2). Therefore, we found the distribution pattern of the pictures in the affective space to be similar to that in the original study with North American participants (Lang et al., 2008), showing that the methodology for remote ratings is sensitive and robust.

**Table 1:** Correlation matrix, valence

**Study**		**Lang et al**	**Lemos et al**
Lemos et al	Spearman’s Rho	0.960	—
	*p*-value	< 0.001	—
Current study	Spearman’s Rho	0.961	0.983
	*p*-value	< 0.001	< 0.001

Lang et al., valence ratings values from Lang et al., (2008) study; Lemos et al., valence rating values from Lemos et al. (2022) study; current study, valence rating values from the present remote study

**Table 2:** Correlation matrix, arousal

**Study**		**Lang et al**	**Lemos et al**
Lemos et al	Spearman’ s Rho	0.898	—
	*p*-value	< 0.001	—
Current study	Spearman’s Rho	0.854	0.917
	*p*-value	< 0.001	< 0.001

Lang et al., arousal ratings values from Lang et al., (2008) study; Lemos et al., arousal rating values from Lemos et al. (2022) study; current study, arousal rating values from the present remote study

The Spearman correlation also revealed that the IAPS ratings obtained in the previous in-person study with Brazilian university students (Lemos et al., 2022) were similar to those obtained in the current remote study. We found a strong correlation between the valence (Table 1, rho = 0.98, *p* < 0.001) and arousal ratings (Table 2, rho = 0. 92, *p* < 0.001). It is also important to note that the IAPS ratings from Lang et al. (2008) and Lemos et al. (2022) were also correlated (see Tables 1 and 2).

### The additional set of food pictures

Spearman’s correlation test confirmed the association between the ratings of the food pictures obtained in this sample and those reported by Lemos et al. (2022). This analysis was carried out to confirm whether, through remote collection of data, it was possible to obtain an additional standardized set of pictures by using the same methodology applied for the IAPS (Lang et al., 2008). The food pictures applied in conjunction with the IAPS pictures were located in the appetitive motivational arm of the boomerang in the affective space (Fig. 3, right panel, mean valence = 6.48, *SD* = 0.41; mean arousal = 3.59, *SD* = 0.39), similar to the findings of Lemos et al. (2022) (Fig. 3, left panel). All food and pleasant pictures from the IAPS were found on the upper arm of the boomerang in both the in-person study conducted by Lemos et al. (2022) (Fig. 3, left panel) and the current remote study (Fig. 3, right panel). This location is thought to represent the appetitive motivational system, while unpleasant pictures were found on the lower arm of the boomerang, which corresponds to the defensive motivational system. We found a correlation between the ratings attributed to food pictures during the Lemos et al. (2022) study and the current remote study, for both the valence (rho = 0.70, *p* < 0.001) and arousal (rho = 0.73, *p* < 0.001) dimensions. This association reinforces the similarity between the in-person and remote studies performed with a Brazilian sample. Hunger did not impact these results since there was no correlation found between the participants’ hunger scores and the participants’ ratings for valence (rho = 0.07, p = 0.26) or emotional arousal (rho =  − 0.06, *p* = 0.33) obtained from food pictures in the current study. Similarly, hunger did not impact valence or arousal ratings in the study of Lemos et al. (2022).

## Discussion

We adapted the in-person methodology recommended by Lang et al. (2008) for use in a remote setting. The reliability and validity of the affective ratings obtained with the remote format in our study are supported by the replication of findings from in-person studies that also applied the normative rating procedure for the IAPS with North American (Lang et al., 2008) and Brazilian (Lemos et al., 2022) samples.

The “boomerang” shape, which has been widely reported in emotion research (Branco et al., 2023), was displayed by the placement of all food and IAPS pictures within the “affective space” in the current remote study. The affective ratings obtained for IAPS pictures in the current study were strongly correlated with the ratings obtained for IAPS pictures in previous in-person studies (Lang et al., 2008; Lemos et al., 2022). In addition, by using the remote format for the normative rating procedure for the IAPS, we succeeded in obtaining a new set of standardized food pictures whose affective ratings correlated with those obtained by a previous study conducted in-person with a similar Brazilian sample (Lemos et al., 2022).

We attribute the success in replicating previous in-person experiments to the following methodological considerations, some of which have also been addressed by Rodd (2024).

1. The participants were informed in advance of the minimal technical requirements needed to complete the experiment, which included access to the task through a computer or notebook, an appropriate Internet connection, and a quiet room.
2. The participants were asked to confirm their availability for the duration of the experiment.
3. We offered clear instructions (written and by video) that followed the recommendations of the original study with North American participants (Lang et al., 2008) as well as practice trials. This approach has been shown to aid in understanding tasks and to reduce participants’ anxiety during remote experiments (Rodd, 2024).
4. The researcher was able to use WhatsApp to address any questions and monitor the experiment.
5. The online instructions and the SAM scale filling process were designed to reproduce the features of the in-person experiment as closely as possible

In a previous study, a digital scale was proposed to measure emotions in response to the online presentation of affective pictures (Betella & Verschure, 2016). This digital scale, called the “affective slider,” was based on SAM. In this proposal, many changes were made in relation to the original IAPS study in terms of both graphic design and instructions. Respondents can select values by dragging a slider through a range, and the starting point of the slider is positioned in the middle of the scale for the valence and arousal dimensions. It has been shown that placing the starting point of a slider (e.g., low, high, right, left, or in the middle of the scale) can bias responses (Bayer & Thomas, 2004). Sliders positioned initially at the midpoint of the scale increase midpoint responses (Sellers, 2013). Establishing the starting point of the slider is even more critical when considering the valence and arousal dimensions. Arousal intensity increases on the SAM from right to left. Thus, the arousal dimension is unidirectional, ranging from calm to alert. The valence dimension is bidirectional, varying from unpleasant to neutral and from neutral to pleasant, with neutral ratings positioned in the middle (Bradley & Lang, 1994). In the current study, unlike with sliders, the cursor’s initial position was arbitrary, and it could be anywhere on the screen to preserve the validity of the emotional aspects evaluated.

The SAM scale has already been widely applied across a wide range of cultural contexts to reliably obtain data about the valence and arousal dimensions of emotion (Barke et al., 2012; Bradley et al., 2001; David et al., 2018; Miccoli et al., 2016; Soares et al., 2015). Therefore, it is highly interesting to apply the SAM scale for studying emotions, especially given that studies using the SAM scale have indicated that the valence and arousal dimensions of emotion are associated with body reactions to emotional visual stimuli (Bradley et al., 2001). The ability to study emotions in larger samples—such as those used in epidemiological studies—is the main advantage of using this method adapted to a remote format. The remote methodology presented here allows epidemiological researchers to obtain data about emotional reactions to a new category of visual stimuli in a diverse and large sample. For instance, the new food pictures applied here can aid in the creation of public health initiatives meant to improve the health of eating environments. Food-induced emotions have a significant impact on consumer decision-making (Gutjar et al., 2015). Consequently, researchers interested in this topic would be able to apply the remote format of the normative rating procedure for the IAPS to a variety of populations.

A limitation of the present study was that the majority of the sample was female. This limitation is explained by the higher percentage of women Brazilian university students (Instituto Nacional de Estudos e Pesquisas Educacionais Anísio Teixeira 2022). In fact, most of the participants were also women in the Lemos et al. (2022) study, which also included Brazilian university students. However, importantly, the affective ratings of both the current remote study and the study by Lemos et al., (2022) strongly correlated with the affective ratings of the study by Lang et al., (2008), in which the sample’s gender distribution was balanced. Another limitation is related to the experimental setup. In order to complete the experiment, the participant must have access to a computer and a private space. This may limit the amount of data collected from low-income individuals who live in multi-person households and/or do not have access to computers. In addition, the participant provides information about the experimental setup, including the size of the computer screen. This may make the information less accurate. However, this is unlikely to introduce systematic bias, although there may be differences between the reported setup and the actual setup.

## Conclusions

In conclusion, the results showed that it is possible to obtain valid affective valence and arousal ratings from a remote experiment conducted using the protocol applied for the IAPS. It was also demonstrated that it is possible to use a remote experiment format to obtain a new standardized set of pictures for use in psychophysiological and/or epidemiological studies.

## Supplementary Information

Supplementary Material 1: The code list of the images from the IAPS catalog and of the food that was used for the experiment.

Supplementary Material 2: Participants’ instructions on the day before the experiment via WhatsApp (second contact).

Supplementary Material 3: Additional instructional pictures were sent to participants via WhatsApp on the day of the experiment (third contact). These pictures were sent along with the didactic video 2.

## Data Availability

All the relevant material supporting the findings of this study are available within the paper or in supplementary material. The didactic videos can be accessed at labnec.sites.uff.br/2023/03/02/experimental_tools/.

## Abbreviations

IAPS: International Affective Picture System
SAM: Self-Assessment Manikin
IQR: Interquartile range
BMI: Body mass index
SD: Standard deviation
WHO: World Health Organization
