# Supplementary material for: Adaptation of the normative rating procedure for the International Affective Picture System to a remote format
Source: Psicol Reflex Crit. 2024 Sep 27;37:41. doi: 10.1186/s41155-024-00326-x (PMC11427625; doi:10.1186/s41155-024-00326-x)
Supplement: Supplementary file 1 — Supplementary Material 1: The code list of the images from the IAPS catalog and of the food that was used for the experiment. [file 41155_2024_326_MOESM1_ESM.docx]

**Supplementary Material 1**

The code list of the images from the IAPS catalog and of the food that was used for the experiment.

IAPS pictures:

The positive pictures included 10 images depicting nature, family, dogs, sports, adventure, and eroticism; the neutral pictures included 30 images depicting objects, people, and landscapes; and the negative pictures included 30 images depicting pollution, disgust, illness, loss, accidents, contamination, animal attack, human attack, and mutilated bodies. List of the 70 IAPS pictures used in the experiments (codes). Negative: 1050, 1220, 1300, 1525, 1930, 2053, 2205, 3060, 3080, 3120, 3130, 3230, 3350, 5961, 5971 5972, 6010, 6020, 9000, 9001, 9220, 9300, 9301, 9320, 9341, 9342, 9440, 9480, 9520, 9903; Positive: 1460, 1710, 2070, 2165, 4660, 4670, 4680, 5260,  5830, 5621; Neutral:1313, 1390, 1935, 1945, 2191, 2210, 2221, 2393, 2850, 2880 , 7046, 7187, 7224, 7247, 7700, 9070, 9080, 9360, 6150, 7002, 7020, 7030, 7034, 7040, 7050, 7052, 7090, 7490, 7705, 7205.

Food pictures:

Unprocessed/ minimally processed foods: watermelon, apple, mandarin juice, salad, corn, egg, lettuce, pear, mango, banana and bean; Ultra-processed foods: gums, potato chips, chocolate bar, ready-to-eat lasagne, ice cream, margarine, cookies, soft drinks, sausages, cookies stuffed with vanilla and Brazilian cheese bread. The pictures were from Lemos et al. (2022) study.
