# Supplementary material for: Adaptation of the normative rating procedure for the International Affective Picture System to a remote format
Source: Psicol Reflex Crit. 2024 Sep 27;37:41. doi: 10.1186/s41155-024-00326-x (PMC11427625; doi:10.1186/s41155-024-00326-x)

**Supplementary Material 2**


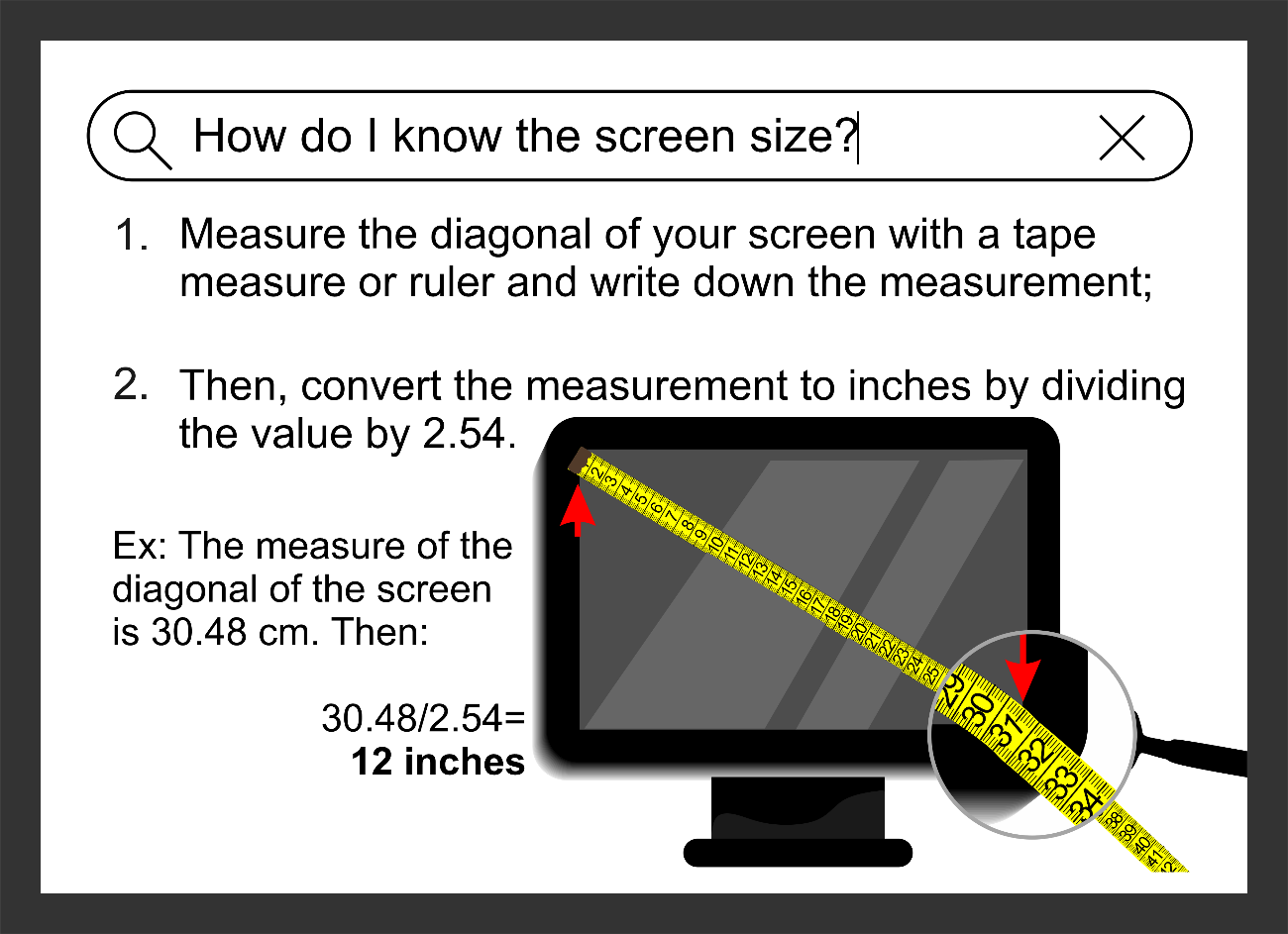
Participants’ instructions on the day before the experiment via WhatsApp (second contact).


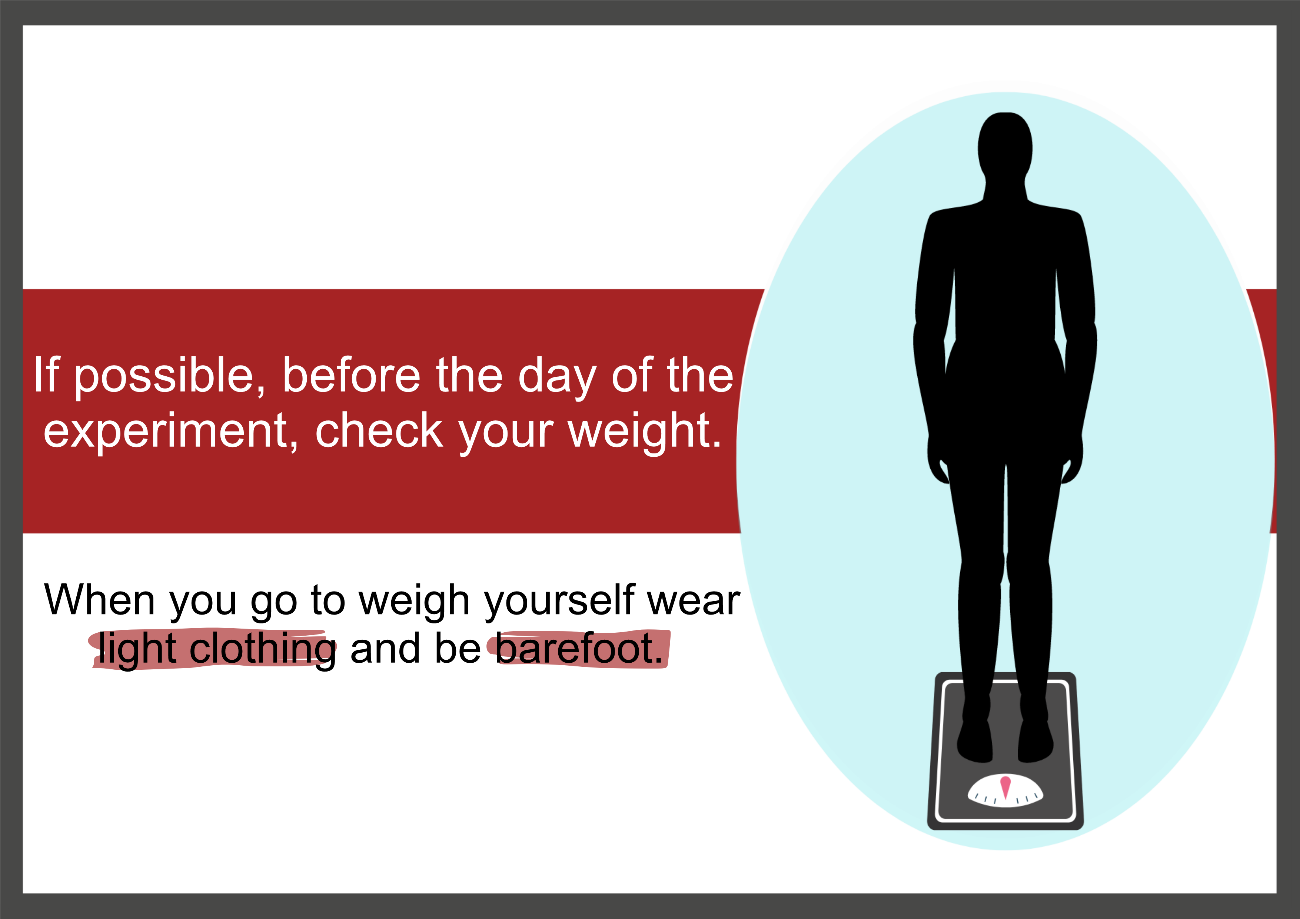

Supplement: Supplementary file 2 — Supplementary Material 2: Participants’ instructions on the day before the experiment via WhatsApp (second contact). [file 41155_2024_326_MOESM2_ESM.docx]
