# Supplementary material for: Adaptation of the normative rating procedure for the International Affective Picture System to a remote format
Source: Psicol Reflex Crit. 2024 Sep 27;37:41. doi: 10.1186/s41155-024-00326-x (PMC11427625; doi:10.1186/s41155-024-00326-x)
Supplement: Supplementary file 3 — Supplementary Material 3: Additional instructional pictures were sent to participants via WhatsApp on the day of the experiment (third contact). These pictures were sent along with the didactic video 2. [file 41155_2024_326_MOESM3_ESM.docx]

**Supplementary Material 3**

Additional instructional pictures were sent to participants via WhatsApp on the day of the experiment (third contact). These pictures were sent along with the didactic video 2.

**
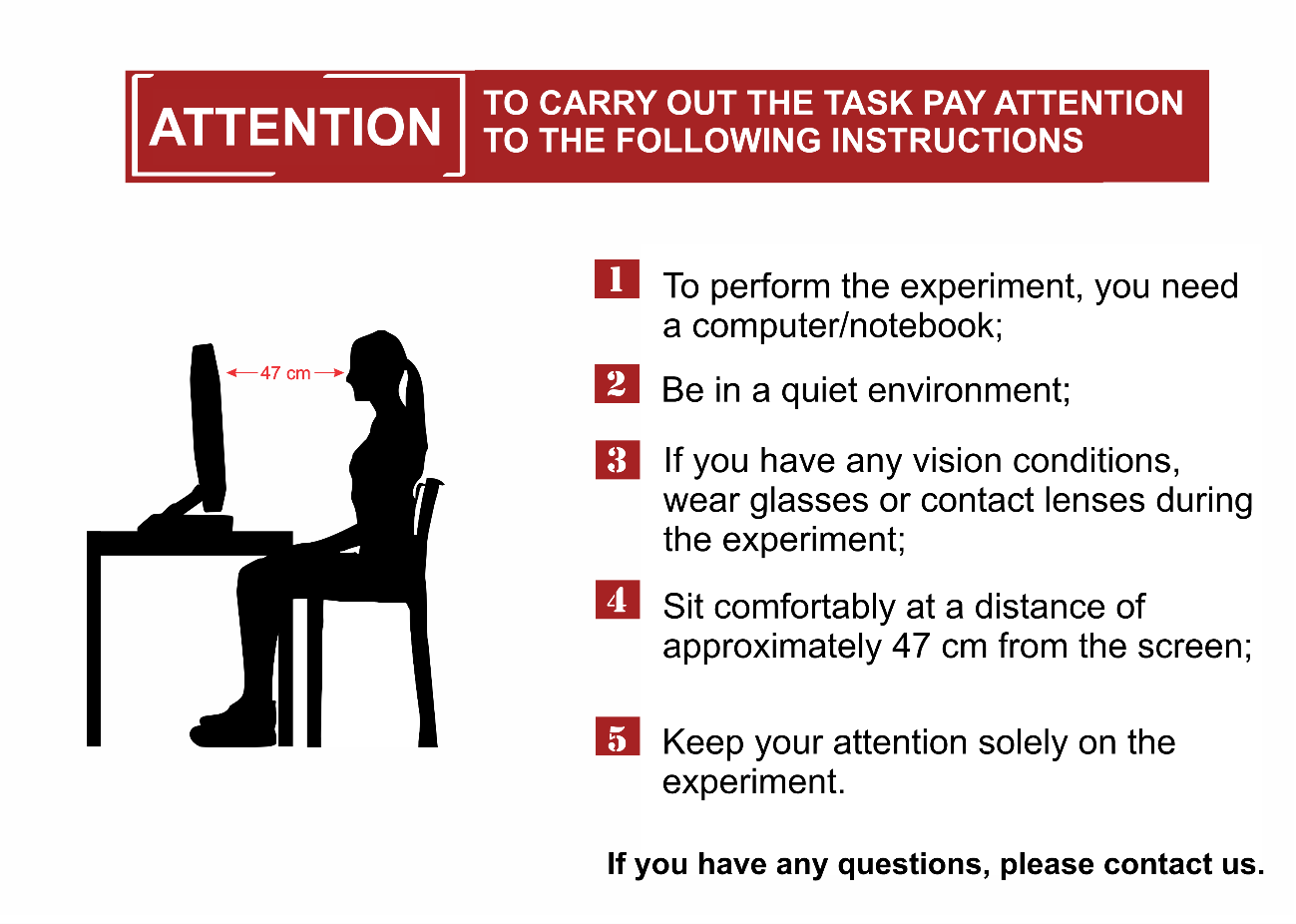
**

**
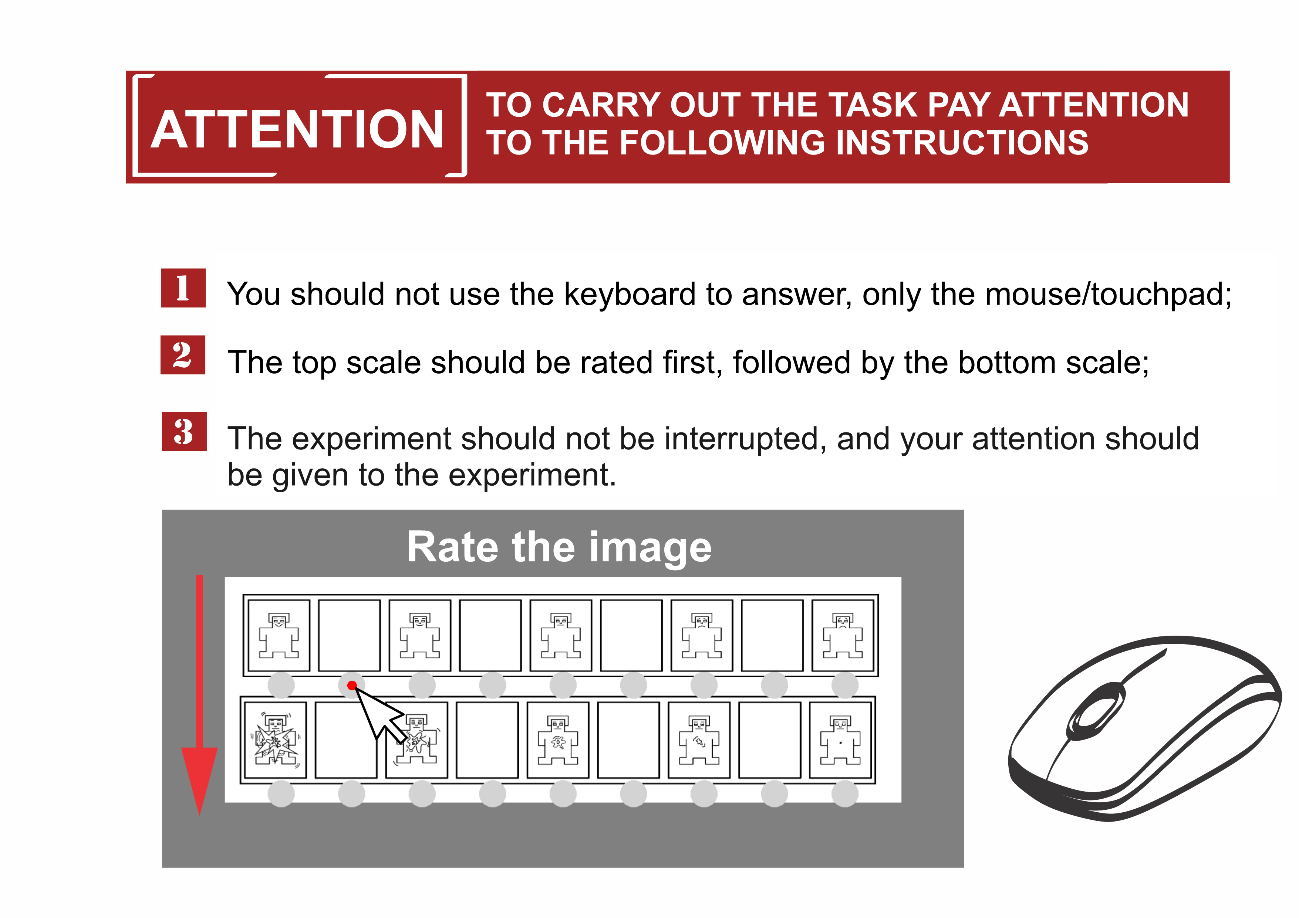
**
